# A systematic review of the barriers and facilitators to lived experience involvement in mental health services

**DOI:** 10.3389/fpubh.2025.1737709

**Published:** 2026-01-23

**Authors:** Selina Shaw, Louisa Shirley, Emma Schroeder, Grace Thompson, Kirsty Watt, Katrina Forsyth

**Affiliations:** 1 School of Health Sciences, The University of Manchester, Manchester, United Kingdom; 2 Greater Manchester Mental Health NHS Foundation Trust, Manchester, United Kingdom; 3 Pennine Care NHS Foundation Trust, Ashton-under-Lyne, United Kingdom

**Keywords:** barriers, facilitators, lived experience, mental health, mental health services, motivation

## Abstract

**Introduction:**

Individuals with lived experience of mental health conditions provide a unique perspective to mental health services. In this systematic review, a broad definition of ‘involvement in mental health services’ was used, which included peer support, peer mentors, and peer specialists. This review aimed to explore the barriers and facilitators reported to peer involvement in mental health services, to develop an understanding of the impact on the ‘peer’ and mental health care.

**Method:**

Five bibliographic databases were systematically searched (inception to May 2024) for qualitative studies, to identify the barriers and facilitators of peer involvement in mental health services. Data were analysed using the COM-B (capabilities, opportunities, motivation, and behaviour) model and Theoretical Domains Framework (TDF) to provide a theoretical framework for understanding the behaviour of being involved in mental health services.

**Results:**

The thirty-three studies included in this review provided data across all components of the COM-B model and eleven of the TDF domains. Barriers included the wider staff teams’ lack of knowledge about the peer role, which impacted the peers’ capability to be involved. The conflict between the professional peer role and identity impacted peers’ motivation, positively and negatively, to remain involved. A lack of social support led to peers feeling stigmatised and excluded from the wider team. When peers felt supported, they could use their skills and lived experience knowledge to drive change in the system.

**Discussion/conclusion:**

This review provides insight into the barriers and facilitating factors experienced by individuals in peer involvement roles within mental health services. The wider implications challenge the notion that peer involvement reduces stigma and discrimination. This review highlights that peer involvement can sometimes increase peers’ experience of stigmatisation, specifically when involved in mental health services.

## 1 Introduction

Individuals with lived experience can contribute uniquely to transform service design and delivery (1). For the purposes of this systematic review, an individual with lived experience refers to someone who has previously accessed mental health services as a service user. Lived experience also includes individuals who have not engaged with formal mental health services or who define their experiences outside of clinical contexts. While these perspectives are important and valuable, they are beyond the scope of this review. This broad operational definition includes volunteers, peer support, expert by experience and individuals involved in co-production. In mental health services, such individuals are often involved under wider frameworks like peer involvement, public and patient involvement (PPI) and volunteering, which can overlap but have distinct characteristics. For consistency in this review, these roles are grouped under the term ‘peer involvement’, used here as an umbrella term for involvement of individuals with lived experience across diverse roles. While ‘peer support’ is commonly used to describe formalised, trained and sometimes paid roles, the included studies in this review used the term ‘peer’ more broadly to encompass voluntary, informal and co-productive roles. To clarify how lived experience is applied in practice, the key roles within peer involvement are now defined.

A volunteer chooses to give their time, often unpaid, to benefit others (2). Volunteering responsibilities can include befriending, mentoring, and giving advice. In mental health services, peers often act as volunteers, offering informal support to current service users by drawing on their own experiences with clinically significant mental health challenges (3). Although peer support increasingly includes formally certified and paid positions, this review focuses on voluntary and informal peer roles represented in the included studies. Peer support draws on techniques and tools, such as insight and their unique skill set developed from their personal journeys (4). ‘Experts by experience’ contribute their knowledge, skills, and abilities, gained through their own mental health journeys (5). A collaborative approach to working and involving individuals is co-production. Co-production involves working in equal partnership with users of health and care services, at the earliest stages of service design, development, and evaluation [(6), p.1]. Together these roles represent diverse ways in which lived experience is used to support others and influence mental health services.

Peer involvement offers a unique perspective to health and care policy, service design and delivery (7). In mental health services, peer involvement plays an integral role in transforming mental health care, by influencing policy and driving change (1). Globally, peer involvement has become a driving force for advocacy and raising awareness of mental health, with peers integrating into various mental health services in different capacities (8). Findings from the Lancet Commission on ending stigma and discrimination in mental health (9), illustrate that peer involvement is a key driver of change to reduce stigma and discrimination toward people with mental health conditions. The commission recommends that peer involvement should play a role in co-designing and co-producing initiatives to reduce mental health stigma.

While peer involvement plays a key role in transforming mental healthcare, there are still apparent challenges to peer involvement in mental health services. Peers are often sought for their expertise and knowledge of a service; however, limited progress has been made regarding remuneration for their expertise. Sartor (1) highlights that peers are expected to be involved in service design and delivery, without compensation for their expertise or service. The lack of remuneration could disempower peers and create a divide between paid staff and unpaid peers (1). Despite initiatives from mental health services to include peers for their valuable contributions, peers are often excluded from research, policy making and organisational leadership roles (10). Additionally, Sunkel and Sartor (11) highlight that negative stereotypes and societal stigma toward individuals with mental health conditions are still apparent. These broader societal attitudes and stereotypes may sometimes influence perceptions within mental health services. This could have an impact on how peers are involved in services and the opportunities that they are able to contribute to (11).

Despite the awareness of the benefits and challenges to peer involvement, most of the existing research has explored barriers and facilitators of involving volunteers who do not have lived experience of accessing mental health services. Barriers to volunteering in mental health services included a lack of role clarity due to the voluntary role not being defined (12). Additionally, volunteers felt that there was no differentiation between their role and other staff, which left volunteers feeling depreciated and undervalued (13). Additional barriers to volunteering, identified by Southby et al. (14), were a lack of time to commit to volunteering, difficulties covering travel expenses, the effort required to volunteer, and a lack of desired skills required in the voluntary role.

In contrast to some of the barriers indicated for volunteers without lived experience, in mental health services, there are also facilitating factors. Research has shown that volunteers can positively contribute to the service user experience and boost staff morale, which facilitates their involvement (15). Volunteers have varied responsibilities, including providing companionship to service users and completing practical tasks, which freed up other staff members’ time to prioritise clinical care. By developing knowledge of the service they were volunteering in, volunteers gained self-confidence in their role (16). A key incentive for volunteering was the sense of belonging connected to their volunteer identity, and the acceptance that they received from other staff in the service (17). Gray and Stevenson (17) also highlighted the positive benefits for the volunteer, such as increased self-esteem, resilience, and a sense of purpose. Volunteering offers the opportunity to gain new experiences, additional skills and potentially progress in the role, with career development cited as a key motivation for volunteers. Personal growth, feeling valued, appreciated and having the opportunity to contribute meaningfully to the service, was also a facilitating factor for volunteers (18).

The research highlights some of the benefits and challenges already known regarding peer involvement. These include peer involvement playing an integral role in influencing policy, driving change, and reducing both stigma and discrimination toward individuals with mental health conditions (1, 9). The challenges to peer involvement included limited progress regarding remuneration for peers’ expertise (1), the exclusion of peers in research, policy making, and organisational leadership roles (10), and the negative prejudice apparent in society toward individuals with mental health conditions (11).

As well as the known benefits and challenges to peer involvement, research on volunteers who do not have lived experience of accessing mental health services, provides context on the barriers and facilitators encountered. These volunteers are individuals who have not personally accessed mental health services but note that they face barriers such as a lack of clarity of role, and insufficient training in mental health services (12, 14). In contrast, facilitators for volunteering included personal growth such as increased self-confidence, self-esteem, and skill development (16, 17). By comparing these factors with those experienced by peers with lived experience, we can identify shared barriers and facilitators, as well as challenges that are unique to peer involvement. This may help to target specific implications for practice and policy, and lead to being more informed of the most appropriate ways to integrate peer involvement in mental health services.

Guided by the SPIDER framework (19), the aim of this review is to understand the barriers and facilitators to peer involvement in mental health services from the peer perspective. This review focuses on qualitative and mixed-methods studies, centring on qualitative studies to authentically represent peers’ lived experiences and challenges associated with peer involvement in mental health services. Although quantitative studies have produced valuable evidence regarding the outcomes, prevalence, and effectiveness of peer involvement in mental health services, they are less able to capture the subjective meanings and contextual factors that shape peers’ experiences. A qualitative approach enables a deeper understanding of the barriers and facilitators to their involvement in mental health services, as perceived by the peers themselves. To execute this, this review used the COM-B model: capabilities, opportunities, motivation and behaviour and the Theoretical Domains Framework (TDF) (20, 21). This specific theoretical framework has been used in this review because both the COM-B model, and the TDF provide a structured and comprehensive framework to ensure that all factors that influence behaviour (i.e., peer involvement in mental health services) are considered. The framework helps to identify if there are specific factors related to peer involvement that could be targeted for intervention, such as a lack of capability, opportunity, and/or motivation (21). The TDF breaks the behaviour down into 14 specific theoretical domains, to provide a further detailed understanding of the factors influencing peer involvement, and can help identify what specifically might need addressing, for example providing more training (capability) to peers, and staff (20).

## 2 Method

This review was pre-registered on the International Prospective Register of Systematic Reviews (PROSPERO) following initial scoping searches but prior to the formal review (registration number: CRD42024506702). This review was reported in accordance with Preferred Reporting Items for Systematic Reviews and Meta-analyses (PRISMA) guidelines (22).

### 2.1 Inclusion and exclusion criteria

Inclusion criteria were informed by the SPIDER framework (19). Studies were included if they were:

(a) Primary peer-reviewed journal articles published in English, or with an English-language full text available.
(b) Sample (S): The population included adults aged eighteen and over.
(c) Phenomenon of Interest (PI): For this systematic review, a broad definition of peer involvement in mental health services was used, which included peer support, expert by experience, volunteers and co-production. Responsibilities in each mental health service were not defined, and could include service delivery, development, and leadership (11).
(d) Design (D) and Research Type (R): Qualitative studies were included in this review. The qualitative data from mixed-methods studies was also included.
(e) Evaluation (E): Studies reporting peers experiences (i.e., perceptions, barriers, or facilitators) related to involvement in mental health services were included.

Studies including the following were excluded:

(a) Participants under the age of eighteen.
(b) Services that were not related to mental health.
(c) Quantitative studies without a qualitative component.

### 2.2 Search strategy

The search strategy was informed by the SPIDER framework (19). The following databases were searched; EMBASE, MEDLINE, PsycINFO, Web of Science and CINAHL Plus from inception to 31st May, 2024. Search terms were developed to capture the sample (peers), phenomenon of interest (peer involvement in mental health services), design and research type (qualitative and mixed-methods studies) and evaluation (barriers and facilitators) (seen in Table 1). A secondary backward citation search was conducted by examining the reference lists of included studies to identify additional relevant data (23). Studies identified through this method were screened using the same screening process and inclusion criteria as the database searches.

**Table 1:** Search terms.

Sample (S)	Phenomenon of interest (PI)	Evaluation (E)	Design (D) and research type (R)
Volunteer* OR voluntary work* OR unpaid work* OR lived experience*OR expert by experience* OR PPI* OR patient public involvement* PPIE* OR Patient and Public Involvement and Engagement* OR nonprofessional volunteer* OR peer support*OR carers* OR service users*	mental health* OR mental illness* OR mental disorder* OR mental health service* OR mental health charity* OR mental health care* OR psychiatric service* OR psychiatric care* OR psychiatric organisation* OR mental health organisation*	Motivation OR motives OR motiv* OR Satisfaction OR reason OR decision making OR opinion OR attitude OR experience OR benefit* OR barrier OR negative OR challenge OR difficult OR positive OR facilitators	Qualitative OR qualitative analysis OR qualitative research OR Grounded theory OR Thematic analysis OR IPA analysis.

The asterisk (*) was used as a truncation symbol to capture multiple word endings and variations of the same root term.

### 2.3 Study selection

The search results from each database were exported and uploaded onto Rayyan for screening (24). Rayyan is software designed to conduct and coordinate systematic reviews (24). Duplicates were identified and removed. First author (SS) screened all search results by titles and abstracts initially against the inclusion and exclusion criteria (stage 1). The articles were screened into three categories; include, maybe, and exclude (with a reason). Full texts of the included articles from the initial screening were screened again (stage 2) using a ‘screening and selection tool’ developed by SS, in line with guidance from Cherry et al. (25). Two reviewers, GT, and KW each independently screened a random 25 % of all articles by title and abstract, and all the full text articles. All decisions were made and logged via Rayyan. Any disagreements between reviewers were resolved by discussion until a consensus was reached. Fleiss’ Kappa (26) was calculated to determine the inter-rater reliability between the three screeners (SS, GT, and KW) for stage 1 of the screening. Fleiss’ Kappa demonstrated that there was moderate agreement, in line with Landis and Kocks’ (27) interpretation, between the three raters, *κ* = 0.588, (95% CI, 0.536 to 0.640), *p* < 0.001. There were no disagreements between the three raters relating to those papers selected for inclusion in the full-text search.

### 2.4 Quality appraisal

Quality was assessed using the Critical Appraisal Skills Programme (CASP) for qualitative research (28). The CASP is a widely used 10-item quality appraisal tool for qualitative research.

The ten checklist items are presented in Table 2 for each of the included studies in this review. As the CASP does not provide a scoring system for the checklist (29) a numerical scoring system was developed and utilised for each item; ‘no’ equals 0, ‘cannot tell’ equals 0.5, and ‘yes’ equals 1, with a maximum score of 10. As suggested by Butler et al. (30) the methodological quality using the total CASP score was categorised using three quality cut-off scores; ‘high’ (> 8–10), ‘moderate’ (6–7) or ‘low’ (≤ 5). Twenty-five percent of the CASPs were independently reviewed between second authors (GT and KW).

**Table 2:** Quality of the included articles, as rated using the CASP for qualitative research.

Study	Q1	Q2	Q3	Q4	Q5	Q6	Q7	Q8	Q9	Q10	Total score
Ben-Dor I et al. (35)	1.0	1.0	0.5	1.0	0.5	0.5	1.0	1.0	1.0	1.0	8.5
Brenisin et al. (76)	1.0	1.0	1.0	1.0	1.0	0.0	0.5	0.5	1.0	0.5	7.5
Simpson et al. (52)	0.5	1.0	1.0	1.0	1.0	0.0	0.5	1.0	1.0	1.0	8.0
Vandewalle et al. (45)	1.0	1.0	1.0	1.0	1.0	1.0	1.0	1.0	1.0	1.0	10.0
Reeves, et al. (41)	1.0	1.0	0.5	1.0	1.0	1.0	1.0	1.0	1.0	1.0	9.5
Janoušková et al. (33)	1.0	1.0	0.0	0.0	0.5	0.0	0.5	0.0	1.0	0.5	4.5
Kessing (77)	1.0	1.0	1.0	1.0	1.0	1.0	1.0	0.5	1.0	1.0	9.5
Storm et al. (51)	1.0	1.0	1.0	1.0	1.0	0.0	1.0	1.0	1.0	1.0	9.0
Chisholm and Petrakis (78)	1.0	1.0	1.0	1.0	1.0	0.0	1.0	1.0	1.0	1.0	9.0
Ehrlich et al. (50)	1.0	1.0	0.5	0.5	0.5	1.0	1.0	1.0	1.0	1.0	8.5
Oborn et al. (79)	1.0	1.0	0.5	1.0	1.0	0.0	0.5	1.0	1.0	1.0	8.0
Gillard et al. (37)	1.0	1.0	1.0	1.0	1.0	1.0	0.5	1.0	1.0	1.0	9.5
Berry et al. (72)	1.0	1.0	1.0	1.0	1.0	1.0	0.5	0.5	1.0	1.0	9.0
Tang et al. (46)	1.0	1.0	1.0	1.0	1.0	1.0	1.0	1.0	1.0	1.0	10.0
Griffiths and Hancock-Johnson (43)	1.0	1.0	1.0	1.0	0.5	0.0	0.0	0.5	1.0	1.0	7.0
Holley et al. (80)	0.5	0.5	1.0	1.0	1.0	0.5	0.0	1.0	1.0	1.0	7.5
Cleary et al. (38)	1.0	1.0	1.0	1.0	1.0	0.0	0.5	1.0	1.0	1.0	8.5
Rebeiro Gruhl et al. (42)	1.0	1.0	1.0	1.0	1.0	0.0	1.0	1.0	1.0	1.0	9.0
Dyble et al. (44)	1.0	1.0	1.0	1.0	1.0	1.0	1.0	1.0	1.0	1.0	10.0
Beveridge et al. (81)	1.0	0.5	0.5	1.0	1.0	0.0	0.5	0.5	1.0	1.0	7.0
Wyder et al. (82)	1.0	1.0	1.0	0.5	1.0	0.0	0.5	1.0	0.5	1.0	7.5
Kivistö et al. (73)	1.0	1.0	1.0	1.0	1.0	1.0	1.0	1.0	1.0	1.0	10.0
Pérez-Corrales et al. (48)	1.0	1.0	1.0	1.0	1.0	1.0	1.0	1.0	1.0	1.0	10.0
Cabral et al. (40)	1.0	1.0	1.0	1.0	1.0	0.0	1.0	1.0	1.0	1.0	9.0
Debyser et al. (47)	1.0	1.0	1.0	1.0	1.0	0.5	1.0	1.0	1.0	1.0	9.5
Soronen (83)	1.0	1.0	1.0	1.0	1.0	1.0	1.0	1.0	1.0	1.0	10.0
Moran (49)	1.0	1.0	0.5	1.0	1.0	0.0	1.0	1.0	1.0	1.0	8.5
Hancock et al. (34)	1.0	1.0	0.5	1.0	1.0	0.0	0.5	0.5	1.0	1.0	7.5
Debyser et al. (84)	1.0	1.0	1.0	1.0	1.0	1.0	1.0	1.0	1.0	1.0	10.0
Poremski et al. (53)	1.0	1.0	1.0	1.0	1.0	1.0	1.0	1.0	1.0	1.0	10.0
Gray et al. (85)	1.0	1.0	0.5	1.0	1.0	0.0	1.0	0.5	1.0	1.0	8.0
Gillard et al. (39)	1.0	1.0	0.5	1.0	1.0	0.0	0.0	1.0	1.0	1.0	7.5
G. et al. (36)	1.0	1.0	1.0	0.5	1.0	0.0	1.0	1.0	1.0	1.0	8.5
Q1: Was there a clear statement of the aims of the research? Q2: Is a qualitative methodology appropriate? Q3: Was the research design appropriate to address the aims of the research? Q4: Was the recruitment strategy appropriate to the aims of the research? Q5: Was the data collected in a way that addressed the research issue? Q6: Has the relationship between researcher and participants been adequately considered? Q7: Have ethical issues been taken into consideration? Q8: Was the data analysis sufficiently rigorous? Q9: Is there a clear statement of findings? Q10: How valuable is the research? Key: Each scoring: no equals 0, cannot tell equals 0.5, and yes equals 1. Total score column: for total score and methodological quality: high (> 8–10), moderate (6–8) or low (≤ 5).											

### 2.5 Data extraction and analysis

Data extraction was completed by author SS, with 25 % being independently reviewed between second authors (GT and KW). The following aspects of the data were extracted into an Excel sheet: (1) general study information, (2) study characteristics, and (3) participant characteristics. Included studies represented a wide range of responsibilities within peer roles, and while some studies reported specific activities and contexts, this information was not consistently available across all studies. Therefore, the review focuses on barriers and facilitators common to peer involvement overall, rather than comparing specific roles.

Data were synthesised using thematic synthesis, deductively guided by the COM-B model (capabilities, opportunities, motivation, and behaviour) (21) and Theoretical Domains Framework (TDF) (20). The entire results section from each study were imported into NVivo 15 for line-by-line coding. Only participant quotes were extracted as primary data, and only those relating to the peer perspective were included. It is acknowledged that participant quotesare interpreted through the lens of the original authors and coding them in isolation may not capture their full context. To address this, quotes were first coded independently to highlight peers’ lived experiences, followed by inclusion of the authors’ interpretations to provide context and enhance understanding, ensuring that peers’ perspectives remained central. Deductive mapping to the COM-B model and the TDF, combined with team discussion and reflection, provided a systematic analytic approach.

Participant quotes were first deductively coded by mapping them onto relevant COM-B model components and associated TDF domains to ensure theoretical alignment while preserving meaning and context. Following this, inductive coding was conducted on both participant quotes and accompanying author interpretations within each COM-B model component and TDF domain to develop sub-themes that added contextual depth. These sub-themes were refined through team discussion and critical reflection.

### 2.6 Theoretical framework

The aim of the COM-B model is to provide a structured theoretical framework to understand and analyse what factors influence behaviour (21). The model integrates three components (capability, opportunity, motivation), which interact and lead to the execution of a behaviour. Capability refers to the individual’s psychological and physical capacity to engage in a specific activity and includes skills and knowledge. Opportunity refers to external factors (outside of the individual), that can prompt or enable a behaviour. This included opportunities provided by the environment (time, location, resources), and opportunities because of social factors, such as social norms and cues. Motivation refers to the internal processes which influence decision-making and behaviour. Motivation included reflecting on experiences to evaluate what had happened and plan future actions (reflective motivation), as well as internal processes such as desire and impulse, which drive behaviour (automatic motivation) (21). While peer involvement encompasses a broad range of roles and responsibilities within mental health services, the COM-B model was used to categorise barriers and facilitators across capability, opportunity and motivation. This provided a structured lens even for these complex and varied behaviours. Specific behaviours differed across the types of involvement and context within the service, but the framework allowed for systematic mapping across all of the varied roles. In this review, the COM-B model helped to develop an understanding of the components that influence peer involvement in mental health services (behaviour), specifically the barriers and facilitators related to each component of the model.

**Figure 1:**
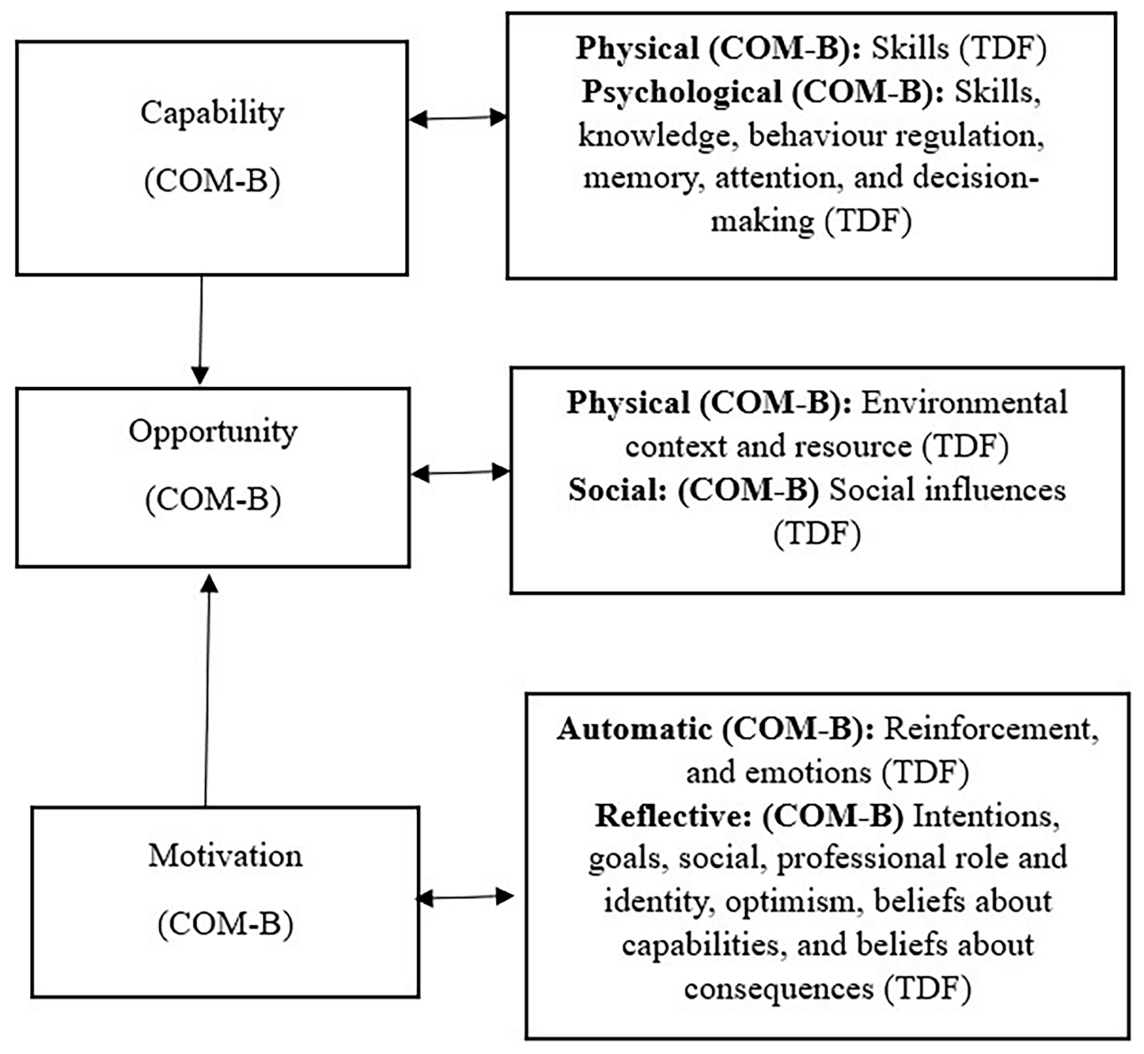
Theoretical domains framework (TDF), mapping onto the COM-B model.

Similarly to the COM-B model, the TDF is a valid integrative theoretical framework primarily used in behaviour change research (20). The framework further explores the psychological, social, and environmental factors that influence behaviour. The TDF expands and can be mapped onto the COM-B model, by breaking behaviour down further into fourteen theoretical domains. This level of detail supported a systematic mapping of the factors shaping peer involvement in its varied form, providing a structured analysis of complex behaviours. It also provided additional context to the factors influencing behaviour, and the specific barriers and facilitators in each of the COM-B model components (20) (see Figure 1 for the COM-B model and TDF domains). In this review, this allowed an understanding of which components and theoretical domains are impacted by peer involvement (behaviour). Other systematic reviews that have used the COM-B model and TDF as a theoretical framework have found that it offers a systematic, structured, and replicable methodology (31).

### 2.7 Researcher reflexivity

General reflections of the study design, process and analysis were collated and shared by the first author (SS) which helped to be transparent about decision-making processes and allowed for a collaborative approach with other members of the research team. All authors had research experience, with professional experience working with individuals with lived experience, in similar roles to those reported in this review. None of the authors have lived experience of mental health recovery or peer support. To minimise bias of experiential attitudes relating to barriers and facilitators, the data was reviewed regularly through reflective discussion with all authors, at each stage of the analysis process. Aspects of the screening, quality appraisal and data extraction were independently reviewed. It was helpful to have multiple authors review aspects of the study process to elicit discussion, reduce bias, and ensure consistency throughout.

## 3 Results

### 3.1 Study design, methodology and participant demographics of included studies

**Figure 2:**
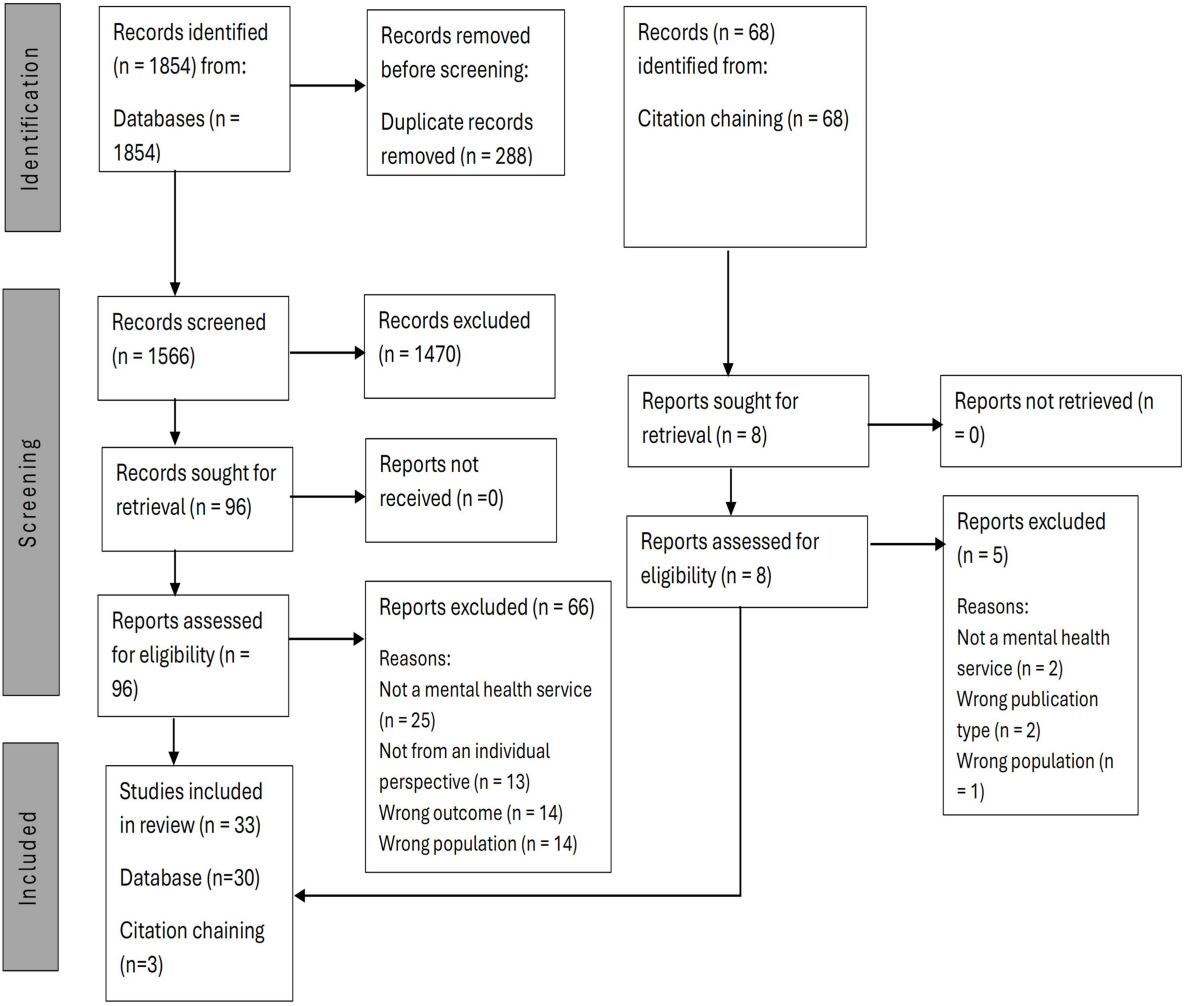
Study selection, PRISMA flowchart.

Eligible papers included thirty-three studies (see Figure 2 for PRISMA diagram). These studies aimed to explore peer involvement in mental health services (as defined in the introduction). Most studies collected data using interviews (*n* = 23) and analysed data using thematic analysis (*n* = 17). 10 of the studies were conducted in the UK, and a considerable proportion in Australia (*n* = 8) (See Supplementary material for included study design and methodology details).

Total number of participants varied, with a range of 2 and 44. There were more female participants (*n* = 183), compared to male participants (*n* = 109). Neither educational level nor diagnosis were specified in most studies (*n* = 25, *n* = 27 respectively) (See Supplementary material for complete participant characteristics).

### 3.2 Key themes identified and mapped onto the COM-B and the TDF

**Figure 3:**
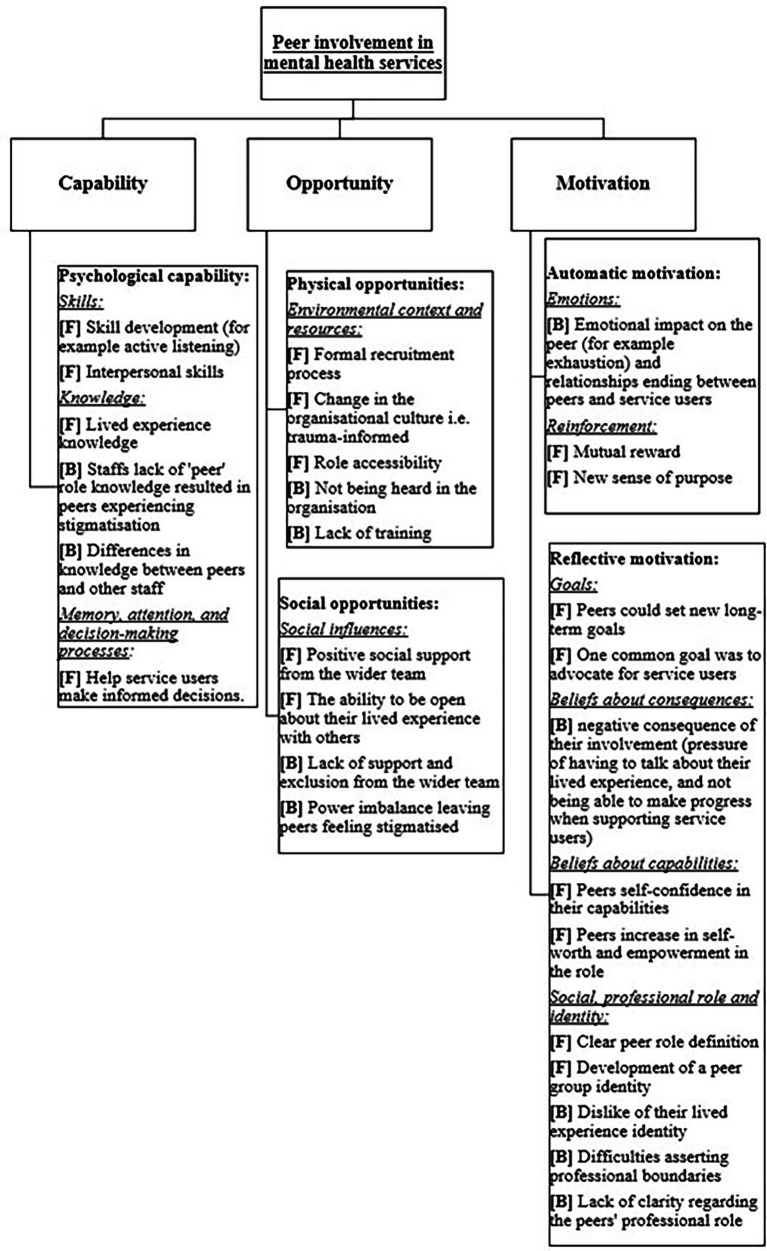
Summary of barriers [B] and facilitators [F] to peer involvement in mental health services, mapped to the COM-B model and theoretical domains framework (TDF).

Data from the included studies, were mapped onto all components of the COM-B model, (21) and eleven out of the fourteen theoretical domains of the TDF (20). Included studies represented a wide range of peer roles, and while some reported specific activities and responsibilities, this information was not consistently available. Therefore, findings are presented across peer involvement rather than by specific role. The three domains from the TDF that were not identified were behaviour regulation (habits), intentions, and optimism. Physical skills were also not identified. The way in which these themes mapped onto the model is described below, and accompanying quotes are presented in text and Tables 3, 4 (see Supplementary material for a matrix of COM-B and TDF themes within each study, which explicitly maps each included study to the relevant component and domain). A summary of the main findings can be seen in Figure 3.

**Table 3:** Table of barriers, sub-themes and participant quotes.

COM-B	TDF	Sub-theme	Quote
Psychological capability	Knowledge	1. Lack of knowledge from other staff members	“I, for example, am not exposed in our service. We ourselves know – among the colleagues who are consumers-providers and do not tell the rest of the staff. There is a stigma and lack of knowledge among staff. Many times, the staff refers to service users as “they,” and we sit and exchange looks with each other [meaning that] we are like you, we are the same, not “they or them.” [(35), p. 7]
		2. Difference in knowledge	“If I’m in that meeting and I say something that is not, sort of psychodynamic, it might be a kind of practical idea, then … it’s never like criticised, but I just feel that, it’s not the way things are done.” [(36), p. 7]
Physical opportunity	Environmental context and resources	1. Interaction with the environment, and not being heard	“I’m listening to peer workers who I’m working beside, who are distressed because of comments made in the workplace or them not getting referrals to their service because they are deemed not important enough.” [(41), p. 173]
		2. Lack of resource available (i.e., training)	“I have not taken any training. I’m not even aware that it’s been offered up where I am. I do not have a policy degree as a social worker or anything. I’m coming from the centre where I was formerly a volunteer and that was it. So it’s lived experience plus I’ve been sent for crisis intervention, conflict resolution these kinds of things. I did that on my own time, she’s [manager] not interested in that. And to me, that is being a peer support worker. But it had no validity to anyone except me and the person.” [(42), p. 6]
		3. Lack of change within the organisational culture	“[because of the] insecurity and uncertainties in the team members, I felt that I had to slow down even more to basically maintain a positive relationship without exacerbating a fragile feeling that is already there in the team” [(72), p. 244]
		4. Lack of structure and implementation of the lived experience role within the organisation	“It [the lived experience role] was completely open, which is great, but also terribly frightening, because you had to learn how to use your personal experiences, and precisely there you do not have any defined tasks and that is not easy when you are new in a workplace. Normally, well, people have some sort of routine in their work. At 11 o’clock, this needs to be done, and then at 12 o’clock there is this. You do not have that as a peer worker. Also, you wonder about ‘how do my colleagues see me?’. There are so many people who struggle with the question of equality. ‘Does my opinion count just as much as the others’?” [(77), p. 37]
Social opportunity	Social influence	1. Lack of social support (leading to stigmatisation, lack of power, and feeling misunderstood)	“If the peer worker is not supported in the right way, they can just become another member of the team, and that power imbalance and the lack of compassion and empathy can become a thing, because they have not had the support.” [(41), p. 173] “I seem to be the only one that’s working in this pure peer role and that has, on occasions, felt a bit lonely.” [(72), p. 244] “One particular member of staff, that has made things difficult, they would not allow me into the office.” [(43), p. 318]. “In the psychiatric hospital, I feel partially stigmatised. I am seen as a former patient and often feel that I am not being told everything” [(33), p. 1426].
		2. Social influences on the individual with lived experience to not share their lived experience of mental health	“We had a manager then who did not quite understand when I actually spoke to him and said ‘look, this is what a [peer worker] is’ he said ‘well I would not tell the team that you have mental health issues.’” [(39), p. 8]
Automatic motivation	Emotions	1. Emotions related to ending the working relationship with the service user	“I found it very difficult for myself. It was like, how could you leave them (service user) after spending three to four months with them? It was quite difficult. Because of the frequent visits, being with them for a few months, then we need to “break up” when his condition improved. I found it miserable at that moment.” [(46), p. 7]
		2. Emotional impact of the role, including toll on mental health, burnout, and leaving the role	“I thought it would be good to share my experiences, but that was a total disappointment. I felt that during my involvement in a peer group, I was preoccupied with my former suicidal thoughts. Now (after quitting as a peer worker) it is easier for me to manage my emotions.” [(45), p. 383]
Reflective motivation	Beliefs about consequences	1. Consequences of the role and the uncertainty and the impact this may have on self	“Listening and stimulating did not help apparently. To some, it was helpful, but not in the majority of service users. That’s when I thought, I am going to come to a dead end, I will get exhausted. I have to get out of this before I get an aversion of my work.” [(45), p. 383]
		2. Consequence of being forced to share their lived experience	“…the first time I’ve talked about my history of mental health in a workplace context was in the interview for the role. Like, it’s not something that you normally talk about at work” [(50), p. 4] “The impression I was being given was I have to get a whole diagnosis of my mental illness and it was just not sitting with me” [(50), p. 110]
		3. Consequence of peer involvement not requiring training	“I’m a little afraid that we will not get tasks and opportunities anymore if we do not follow a training programme.” [(45), p. 383]
Reflective motivation	Social/professional role/identity	1. Excluded from the wider team (not having the same access and equality as other members of the team)	“I felt like an outsider; nobody spoke to you, nobody in the staffroom spoke to you.” [(52), p. 667]
		2. Hide identity	“The fact that a case-manager can tell you, you cannot self-disclose to this person, you can self-disclose to that person. It’s an issue. Someone with knowledge from experience, it’s something that is completely personal that you are being asked to erase. They’re basically telling me to erase a part of myself. A part that can be very easily found out.” [(35), p. 8]
		3. Lived experience identity	“I’m kind of shedding my service-user shell, even though I’m still a service user” [(44), p. 86] “You have to be on your best behaviour, because if I get upset I’m worried that people are going to think, ‘ooh, she’s having a service user moment’. There’s lot of pressures to kind of gain acceptance.” [(36), p. 6] “I still do not like part of that identity [having lived experience of mental health problems] for peer support when I first went in the team, you could see people looking, thinking the medical model was there, it was like ok, “you are here Peter, you look ok, but what’s wrong with you, I do not wanna be mad to have the job” [(44), p. 87]
		4. Maintaining professional boundaries and the impact on the self	“It has been quite hard to assert myself sometimes and, you know, try and be a professional.” [(36), p. 9] “You build a connection and you try to keep it separate from our own life and it can be a challenge for most people. I do have a psychologist as a therapist to help me separate my life from my professional life.” [(51), p. 5] “I think (service users) were thinking I would help them make complaints, but I wasn’t going to do that because I’m part of the team. Although I’m separate, I’m still a part of the NHS team as well. It’s difficult because I was in the middle. You have all the professionals, then you have the peer, and then you are in between them.” [(52), p. 668]
		5. Not understanding the lived experience role, and the need for education and promotion and progression of the role	“I think one of the greatest challenges of a peer support specialist work is the clarity of role. The clarity of role always comes into question, it’s always in doubt, because it is intangible at times and it’s hard to quantify our outcomes. So it’s not very straight, black and white kind of thing?” [(53), p. 230] “I’m actually quite certain that I want to move on from the role. I think [there are] a lot of considerations. I think no 1. was definitely like the space to grow, I think that was the biggest thing that came up I guess. I felt like I think over the past few months, I kind of realised, like how restricting the role can be and I feel like I want to do something more.” [(53), p. 232]

**Table 4:** Table of facilitators, sub-themes and participant quotes.

COM-B	TDF	Sub-theme	Quote
Psychological capability	Skills	1. Utilising interpersonal skills to develop relationships	“The relationship is being created by the immediate sharing of my life experience; so actually, right away, what is created is a different, less border-like relationship than the professionals have.” [(33), p. 1425].
		2. Utilising life skills	“What do we bring with us? What is it you can do? What kind of competencies do you have? I do not think there’s just one answer. There are just as many answers as there are peer workers, because we all have something different with us. It’s all of our baggage: the illness history, my academic background and that I like to cycle and play basketball.” [(77), p. 38]
		3. Skill development	“Listening is a really important skill to have, before I was always used to throwing out advice, and I really needed to get better at that.” [(34), p. 354]
Psychological capability	Knowledge	1. Knowledge about the impact of lived experience on others	“I think a lot of it is just understanding because sometimes it’s spotting the danger signs. Sometimes it’s just being aware of what might be difficult. I mean, everyone has such different triggers, but there are certain topics that you can go, well, you know, which someone who has not had that experience might not realise because it’s not always the obvious ones. So sometimes it’s a little bit pre-emptive.” [(79), p. 1312]
		2. Insider knowledge and the connection of shared experience (which leads to the start of a trusting relationship)	“you go and try and talk to the individual and then I’m upfront. I say, ‘You know, I’m a service user. I’ve been on the ward like you,’ and their expression immediately turns to delight and they say, ‘Oh, have you! Can you help me?’ And, you know, they immediately make a connection with you” [(37), p. 439].
Psychological capability	Memory, attention, and decision-making processes	1. Helping others to make informed decision	“Help them (service users) explain their position better than they might be able to do if they were just there in a room full of clinicians or the tribunal by themselves without support.” [(38), p. 1270]
Physical opportunity	Environmental context and resources	1. Formal recruitment process	“…it actually recognises that the role is a proper job … if they go through a recruitment process they realise the importance and the responsibility the job brings.” [(39), p. 686]
		2. Physical opportunities to self-disclose (the individual own lived experience of mental health conditions)	“I’ve done it [self-presented my story] with clinicians at [agency name] and also with the medical director. I would get into what the experience is like and have people understand what it’s like to live with a mental health condition and then also what is helpful and what is not helpful from my perspective.” [(49), p. 355]
		3. Keeping the environment the same, but the service user relating more to the individual with lived experience	“…they tell me a lot more things that they do not tell their care coordinator. I’m sure some of them know that we all communicate anyway and we have to write our notes on the computer but it might just be that actually they feel more comfortable telling me certain things.” [(37), p. 440]
		4. It is not all about sharing lived experience (knowing the right time and situation)	“I learned that my knowledge from experience is divided into stories: There is a specific story for each situation, it is not that a service user comes, and you spill it out [i.e., your personal story,]. This is the right way. You must think about where, how much, and why you self-disclose”. [(35), p. 8]
		5. The opportunity to be their authentic self	“For me, it feels like it’s about being human and I do not feel I need to be saying, ‘I’ve had mental health problems, it was tough for a while.” [(79), p. 1313]
		6. Facilitate change within the workplace culture	“I often consult on a lot of their ideas around language, just as a basic example they’ll send me documents and I will read over them and they just want the consumer recovery perspective on them. It’s good because I can change a bit of the language and re word questions to be more suitable.” [(38), p. 1270]
		7. Accessibility to the role, and development	“There were not a whole lot of us [peer workers] around, but in the last year in the [region], it has exploded. We have virtually complete autonomy to create and implement projects. It’s an incredibly welcoming environment for peers.” [(40), p. 109]
Social opportunity	Social influence	1. Good social support, ongoing supervision, and supportive working conditions	“Debriefing because myself and the other peer workers have mental illness. So if we come back from a client visit or if something happens in a group, the team rallies around us peer workers and says, do you want a debrief about this, and the managers as well” [(41), p. 174] “I do feel like I can go to anyone and everyone with any questions and yeah, just ask really, so it’s good…Yeah. I mean, I had a little, I had a little blip a couple of months ago and I had to go into hospital, but they supported me a hundred percent.” [(43), p. 319]
		2. Recognition of the lived experience role, and a shift in the wider group identity	“It feels like they [professionals] appreciate this experiential expertise enormously and what we have gone through in our own lives. And coming into the work community, we are treated as equal.” [(73), p545]
		3. Modelling lived experience roles, reducing stigmatisation and shifting social attitudes	“I speak with my non-peer co-workers daily and encourage them to look at things in a different way, which translates into an opportunity for collaboration on our team.” [(40), p. 108] “It’s [Peer Support] taken the stigma away and also having a job where you do not have to hide it [mental health conditions] people automatically know that you have had problems, it has been emancipating” [(44), p. 86]
		4. Being the social support for service users	“[Recovery] was not a fun time by any means, but then to be able to make even a tiny of a fraction of change to make that a little bit easier for someone else, just makes it just so incredibly worthwhile. It [reinforces] everything that you learn in recovery.” [(81), p. 8]
Automatic motivation	Reinforcing behaviour	1. The experience is rewarding	“So I guess what drives me, I feel like my peer support work is mutually beneficial like I feel like being able to share my life with people to where it’s like, yeah I get that.” [(42), p. 5] “It is also the case that in having contact with patients, you help them in their process for a while, but your own process also continues. Often, seeing how other patients deal with it means that you learn things for yourself as well. So that is a dynamic that goes in both directions.” [(47), p. 565]
		2. The commitment is an incentive to continue being involved	“An obligation, a commitment, knowing that you have to get up at a certain time, it’s like a job, in other words, it gives you stability, commitment, an obligation a sense of normality, as if you work” [(48), p. 6]
Reflective motivation	Goals	1. Advocate for others	“I always stand beside the service user and then see how we can combine the service user’s goals and interests with the interests of the organisation” [(45), p. 383]
		2. Enabled to pursue different goals	“One of the things I would actually really love to do is start, like, these recovery groups that we lead. To make it nationwide” [(49), p. 356]
Reflective motivation	Beliefs about capabilities	1. Being aware of their perceived capabilities	“I cannot recommend medication, because I’m not a doctor, but I can definitively sit in the office and help you advocate about your symptoms and what you want to do moving forward.” [(51), p. 4]
		2. The impact of training in relation to role and self-confidence	“I’m a peer support worker, I’ve got my shiny badge; I’ve been trained.” [(52), p. 666]
		3. Perceived value	“Over time, I’ve realised that when I am saying my opinion, they are really listening to it. It’s like I’m coming up with something that they have not thought of.” [(33), p. 1425].
		4. Empowerment	“From having a completely negative life I have gone to realising, all of a sudden, that I am a totally positive person who helps many people, this is reassuring, you feel good. It gives you so much.” [(48), p. 6].
Reflective motivation	Social/professional role/identity	1. Knowing their professional role, boundaries and identity when working with service users	“Yeah, I played with how much do I share? How much do I like I want there to be a boundary set between I’m still here for you, it’s not about me but I wasn’t there as a stomping ground for my issues.” [(42), p. 7] “There is a degree obviously to which you have to be professional … how you conduct yourself … professional conduct is something that is covered in the training … I do feel like a professional when I’m up there.” [(36), p. 8] “I think I’ve reached this point where I’m quite clear, in fact I’m very clear about my role, like what I’m supposed to do and there’s this basic structure and I think initially it was just like coming up with things because when you are in a new department and they have never had peer support specialist [PSS], they have never heard of it, you actually suggest or initiate right?” [(53), p. 231]
		2. Lived experience group identity	“I’m with the other people I know that are also peer providers, or in recovery and just doing the certified peer specialist training, it really helps to feel just the confidence in myself, that I’m not settling for anything. There is a lot of emotional support in peer groups.” [(49), p. 354].
		3. A new identity that allows individual to make socially valuable contributions	“Do you know what I really like? Being able to tell people that I’m a mental health peer support worker and I work for the NHS, instead of ‘Oh, I’m on (government financial support)’.” [(52), p. 665] “‘It seems as if you are no longer burdened with the illness, it seems like you are no longer ill. It is very gratifying because you completely forget that you are ill, and you feel like a different person. It makes you feel like a person in an important position.” [(48), p. 5].

#### 3.2.1 Capability

The COM-B model proposes that behaviour (i.e., peer involvement in mental health services), requires capability. The TDF expands on the factors that influence capability (20). In the TDF, physical capability includes skills (e.g., development, competence, and interpersonal skills), strength and stamina (32). Psychological capability includes knowledge, skills, behaviour regulation, memory, attention, and decision-making.

##### 3.2.1.1 Psychological capability (COM-B): skills, knowledge, and memory, attention, and decision-making processes (TDF)

###### 3.2.1.1.1 Skills (TDF)

This review identified psychological capability skills as facilitators of peer involvement, including skill development, interpersonal skills and competent use of these in peer roles.

A key facilitator was peers’ ability to apply interpersonal skills, such as listening, being non-judgemental, communicating and sharing their lived experience with service users. This helped peers form relationships with service users, especially when service users perceived peers as ‘different’ and ‘less border-like’ than other staff. ‘Less border-like’ meant service users saw peers as ‘friends’ rather than staff [(33), p. 1425].

Another facilitator of peer involvement was peers’ capability to develop skills. Peers acknowledged that active listening was a skill they needed to improve, as they had previously ‘thrown out advice’ to service users without listening [(34), p. 354, see Table 4]. Developing this skill through peer involvement and training, helped peers engage more effectively with service users.

###### 3.2.1.1.2 Knowledge (TDF)

The TDF indicates that psychological knowledge includes awareness of a specific condition, or a procedure (32). This review identified psychological knowledge related barriers and facilitators to peer involvement in mental health services, including a lack of staff knowledge of peer involvement, differences in knowledge between staff and peers, and lived experience knowledge.

Staff member’s lack of knowledge about peer involvement was a barrier, as some staff were unaware of who the peers were, leading peers to feel stigmatised and unable to disclose their lived experience to others. This created a divide between peers and other staff. Peers felt isolated and stigmatised, limiting their capability to be meaningfully involved [(35), p. 7].

Knowledge differences between peers and staff also acted as a barrier to peer involvement in mental health services. Peers tried to contribute to service meetings using their own knowledge, but it often did not align with service models. Services often relied on specific models and psychological perspectives unfamiliar to peers. This left peers feeling that their knowledge capabilities were not as valid as other individuals in the team, reducing their participation in future meetings [(36), p. 7].

Peers’ own lived experience (‘peer knowledge’) facilitated involvement by helping to build connections and increase engagement with service users, by sharing similar and relatable experiences [(37), p. 439, see Table 4].

###### 3.2.1.1.3 Memory, attention, and decision-making processes (TDF)

The TDF defines memory, attention, and decision processes as the capability to retain, attend to, and choose between different pieces of information (32). Peers valued involvement in decision-making processes as they could help service users make informed decisions, advocate for their rights and help them to navigate complex decisions [(38), p. 1270].

#### 3.2.2 Opportunity

The COM-B model proposes that behaviour (i.e., peer involvement in mental health services), requires opportunity and opportunities can be both physical and social. The TDF provides further details on factors influencing opportunity (20). Physical opportunities include environmental context and resources. These include the peers’ opportunity to develop their abilities, skills, and growth, such as material resource, environmental interaction, and staff culture (32). Social opportunities include social influences, which are factors that can impact and change how peers may think, feel, or behave, such as social pressure, support, power, and social norms (32). These factors may discourage or encourage peer involvement.

##### 3.2.2.1 Physical opportunities (COM-B): environmental context and resources (TDF)

###### 3.2.2.1.1 Environmental context and resources (TDF)

Barriers and facilitators to peer involvement in mental health services, related to physical opportunities, included accessibility to the peer role, opportunities for development, formal recruitment, influence on organisational culture, training, and not being heard by staff.

A facilitator was the opportunity for formal recruitment. Peers indicated that if their involvement included an interview, and a job description it would be recognised by staff as a ‘proper job’ [(39), p. 686]. This formal structure made peers feel their role was valued and important.

Another facilitator of peer involvement was the opportunities peers had to influence the organisational culture. For example, peers challenged how staff used language to describe mental health, and promoted trauma-informed care [(38), p. 1270, see Table 4].

Increased access to the role also facilitated peer involvement. Peers highlighted that mental health services were more welcoming of peer involvement, which enabled peers to have more autonomy and opportunities to implement projects in services [(40), p. 109].

A key barrier was peers’ perception of not being heard in the organisation. This discouraged peers integration into the role and service. For example, some peers would not receive referrals to support service users, and staff sometimes made distressing comments about peers. Peers felt unimportant and that their input was ignored. This led peers to question whether they wanted to continue their peer involvement [(41), p. 173].

Another barrier to peer involvement was a lack of resources, particularly training. Training was often inconsistent or unavailable, and some peers were unaware of any training opportunities. The lack of development left peers feeling devalued [(42), p. 6].

##### 3.2.2.2 Social opportunity (COM-B): social influence (TDF)

###### 3.2.2.2.1 Social influence (TDF)

Barriers and facilitators to peer involvement in mental health services related to social opportunities included a lack of staff support, power imbalances, working conditions and using peer roles to reduce stigma and shift social attitudes.

A key barrier to peer involvement was the lack of social support peers received from staff. Peers reported feeling excluded and unwelcome by staff, with one peer reporting that they were not allowed in communal staff areas [(43), p. 318].

Peers perceived a power imbalance between themselves and staff due to previously being service users. This was a barrier to peer involvement as peers felt stigmatised by staff still viewing them as ‘former patients’ [(33), p. 1426, see Table 3]. Staff sometimes withheld information from peers while sharing it with non-peer colleagues. Related to this power imbalance, some senior director/managerial staff told peers not to disclose their lived experience of mental health problems to colleagues and service users. This left peers feeing unsupported.

Support from staff facilitated peer involvement, including regular check-ins, supervision, and debriefs after incidents. For example, if there was an incident in a group or on a client visit, the team would ‘rally’ around the peer worker to ensure they were ok. This helped peers feel safe and supported by their team [(41), p. 174].

Peers’ openness about their lived experience, with staff and service users, also facilitated peer involvement. This openness helped to reduce the stigma around mental health conditions [(44), p. 86, see Table 4]. Staff knew peers had experienced mental health conditions; therefore, peers did not have to hide their mental wellbeing from staff and service users, and peers felt a sense of freedom in their role.

#### 3.2.3 Motivation

The COM-B model proposes that behaviour change depends on altering capability, opportunity, or motivation (21). Changes in capability or opportunity can influence motivation to engage in behaviours such as peer involvement. Motivation in the COM-B model includes both reflective processes (e.g., planning, evaluating) and automatic processes (e.g., desires, wants and needs) (21).

The TDF elaborates on motivation factors (20). Reflective motivation includes beliefs about consequences, intentions, goals, optimism, beliefs about capabilities and social/professional role and identity. Automatic motivation includes reinforcement and emotions.

##### 3.2.3.1 Automatic motivation (COM-B): emotions and reinforcing behaviour (TDF)

###### 3.2.3.1.1 Emotions (TDF)

The TDF defines emotions as reactions to personally significant events or matters (32). This review identified emotional impacts as a barrier to peer involvement.

Peer roles affected wellbeing and motivation to remain involved. One peer experienced overwhelming emotional distress, suicidal thoughts, and left the role. Peers felt better able to manage their emotions when withdrawing from peer involvement [(45), p. 383]. Others felt tired, drained, and overwhelmed by the number of service users they had to support, leaving them ‘worn-out’.

Another barrier to peer involvement was the emotional impact of ending relationships between peers and service users. When peer support ended, or the service user’s mental health improved, this felt like a relationship ‘break-up’ to peers. Peers perceived they were rejecting or abandoning the service user and described the ending of their peer support as ‘miserable’ [(46), p. 7]. No facilitators relating to emotions were identified.

###### 3.2.3.1.2 Reinforcement (TDF)

The TDF defines reinforcement as the use of rewards, incentives, punishment, and sanctions (32). This review identified only facilitators to peer involvement, such as peers’ perception that involvement was rewarding and incentivising.

Peers described helping and supporting service users as personally rewarding, which reinforced their motivation to remain involved. Peers also reported learning new ways to manage their own mental health [(47), p. 565, see Table 4].

Another facilitator was that peer involvement provided peers with a new sense of purpose. Peers viewed their involvement as a commitment to fulfil, which provided peers with a sense of ‘normality’ through routine and responsibility. Peer involvement offered stability, positively impacting peers’ mental health and wellbeing [(48), p. 6].

##### 3.2.3.2 Reflective motivation (COM-B): goals, beliefs about consequences, beliefs about capabilities, and social, professional role and identity (TDF)

###### 3.2.3.2.1 Goals (TDF)

The TDF defines goals as desired outcomes, including goal setting, prioritisation and action planning (32). Facilitators to peer involvement in mental health services, included peers’ personal goals and their desire to advocate for service users.

Peer involvement helped individuals identify and pursue new goals. Peer involvement was not seen as ‘just a job’, some peers had long-term goals, such as expanding peer involvement nationwide [(49), p. 356].

Another common goal for peers was to advocate and represent services users, particularly in team meetings. Peers aimed to represent service users’ goals and interests, standing beside them to provide a voice [(45), p. 383, see Table 4].

###### 3.2.3.2.2 Beliefs about consequences (TDF)

The TDF defines ‘beliefs about consequences’ as an individual’s understanding or acceptance of behaviour outcomes, such as anticipated regret, and expected positive or negative results (32). This review identified barriers related to peers’ perception of the impact and consequence their involvement could have on themselves.

One barrier identified was peers believing that their involvement had negative consequences. This included peers reaching a ‘dead-end’ when supporting service users, leading to peers withdrawing their support due to feeling ‘exhausted’ and fearing developing a dislike toward their peer involvement [(45), p. 383, see Table 3].

Another barrier was staff pressuring peers to disclose their lived experience. Peers were asked to disclose their mental health diagnosis to clarify their role in clinical care [(50), p. 110]. This caused discomfort and feelings of devaluation when peers were reduced to their diagnosis.

###### 3.2.3.2.3 Beliefs about capabilities (TDF)

The TDF defines ‘beliefs about capabilities’ as self-confidence, empowerment, self-esteem, and perceived competence (32). Facilitators included peers understanding of their role, the impact of training on self-confidence and the empowering nature of peer involvement.

One facilitator to peer involvement was peers’ recognition of their perceived capabilities. For example, they recognised that they cannot recommend medication, as they were not medical doctors, but could support service users in other ways, including advocacy [(51), p. 4].

Training helped peers feel competent and able to apply their learning to the role. This increased peers’ self-confidence and preparedness. Training also legitimised the role, as they now had a title, ‘peer worker’, rather than being referred to as a previous ‘service user’ [(52), p. 666, see Table 4].

Involvement also empowered peers, helping them to recognise their strengths and ability to help and support others, despite past challenges. Helping others by using their lived experience, increased peers sense of self-worth and made them feel ‘good’ [(48), p. 6].

###### 3.2.3.2.4 Social, professional role and identity (TDF)

The TDF defines this domain as behaviours and personal qualities linked to social or professional role, identity or boundaries of an individual in a social or work setting (32). Barriers and facilitators to peer involvement in mental health services included challenges around lived experience identity, professional boundaries, and role clarity.

One barrier to peer involvement was peers’ discomfort of their ‘lived experience identity’, particularly disliking that they had to have a mental health diagnosis to get the job. This highlighted a conflict between peers personal lived experience and their emerging professional identity through peer involvement. Peers also felt pressure to always be on their ‘best behaviour’, and hide distress, fearing staff would judge them as ‘having a service user moment’ [(36), p. 6, see Table 3]. This pressure led to peers’ efforts to appear more professional to gain staff acceptance [(44), p. 87].

Some peers reported finding it difficult to assert professional boundaries, such as not having contact with service users outside of the service. This was a barrier to peer involvement, as peers did not want their professional role to overlap with their personal life. One peer even sought support from a psychologist to manage these boundaries [(51), p. 5].

A lack of role clarity was another barrier to peer involvement. Peers reported that staff would question their purpose, and that it was difficult to ‘quantify’ what their role entailed. Unlike clinicians, the peer role was perceived as unclear and sometimes reduced to an ‘unglamourous assistant’. Other peers described the role as restrictive with no space for growth or progression. This impacted peers’ motivation to remain involved and led to some peers leaving the role [(53), p. 232].

Social, professional role and identity also facilitated to peer involvement. When peers could clearly define their role to staff and service users, it provided structure and strengthened their professional identity [(36), p. 8, see Table 4].

Developing a peer group identity also facilitated peer involvement. Being part of a group gave peers emotional support and increased their sense of belonging. Additionally, the peers’ professional role allowed them to contribute meaningfully and reclaim a valued identity. Make contributions that they perceived to be socially valuable. Peers felt they now had a ‘place and function in society’, challenging the stigma of mental illness and improving their own self-worth (45).

## 4 Discussion

This systematic review examined barriers and facilitators to peer involvement in mental health services using the COM-B model and TDF to explore key behavioural and contextual influences (20, 21). Findings are framed within PPI literature and analysed using the COM-B model and TDF to interpret behavioural factors influencing peer involvement.

A key finding highlights that peer’s capability, particularly within the TDF domain ‘knowledge’ can act as both a barrier and a facilitator to their involvement in mental health services. Staff members’ limited knowledge of the peer role restricted meaningful peer involvement, aligning with broader PPI literature on the undervaluation of experiential knowledge in mental health services, compared to clinical expertise (54). This also reflects concerns in PPI research, highlighting how failing to recognise peers’ contributions in services can lead to an undervaluation of their knowledge and expertise and how experiential knowledge is often developed through sharing lived experience (55, 56). These findings contribute theoretically by illustrating how experiential knowledge impacts psychological capability within COM-B and TDF frameworks, advancing understanding of peer involvement processes.

Notably, this review found that peers’ own lived experience knowledge enhanced their capability to meaningfully engage with mental health services and build connections with service users. This supports Byrne and Roennfeldt (57) who identify lived experience as a distinct form of knowledge that adds value to peer involvement and service provision.

Opportunities, particularly social opportunities, as reflected in the TDF domain ‘social influence’, emerged as both barriers and facilitators to peer involvement. A recurrent barrier was the lack of social support, combined with persistent power imbalances between peers and mental health staff, which contributed to experiences of stigmatisation and discrimination. This reflects broader PPI literature, such as O'Shea et al. (58), which highlights how organisational culture maintains professional dominance by prioritising clinical and managerial perspectives, often overlooking or undervaluing peer voices. Veldmeijer and van Os (59) describe ‘participatory assimilation’ where involvement of individuals with lived experience in projects, such as research and service design, is often mandated by policy, but organisational preconceptions can create further power imbalances and limit meaningful peer participation (59). These power imbalances shape environments where peers feel excluded, stigmatised and unable to fully participate. However, when peers have the physical opportunity to influence staff attitudes and culture, such as advocating for trauma-informed language, meaningful peer involvement is facilitated. This demonstrates the importance of organisational readiness and culture change, as identified in implementation frameworks like (60). This framework emphasises that a supportive and open culture is essential for embedding new roles and practices. These insights contribute theoretically by highlighting how opportunity, within COM-B and TDF, is shaped by organisational power structures and cultural norms, indicating the need for organisational and social change to support ongoing peer involvement.

Motivation, particularly within the TDF domain ‘social, professional role and identity’, reflects the complex, often conflicting nature of peers’ identity as they balance their lived experience with emerging professional roles. As highlighted in the results, peers experienced tensions between their lived experience identity and professional role, including stigma and discrimination from staff, such as pressure to behave in ways that avoided being perceived as former service users, and to be on their ‘best behaviour’ or ‘hide distress’. These tensions may also contribute to ‘peer support drift’ where peer roles shift away from their intended recovery-orientated focus, valued for their uniqueness of lived experience, toward more clinical or medicalised roles due to pressures to confirm within the system (61). This aligns with L et al. (62) which shows that peers experience of stigma can undermine professional identity, cause emotional distress, reduce wellbeing, impair motivation and belonging and lead to exclusion. These findings deepen theoretical understandings of how peers negotiate identity in clinical settings and extend PPI literature by highlighting emotional and social challenges, such as stigma, identity, and boundary ambiguity, including ethical and boundary challenges in peer support, such as dual relationships, negotiating personal disclosure and professional responsibilities (63, 64) that affect meaningful peer involvement.

### 4.1 Critical evaluation of the included studies in this review

The quality appraisal revealed that most included studies were of high methodological quality, with 22 out of 33 studies scoring between 8.5 and 10, and 8 studies achieving full scores on the CASP. This suggests many studies met CASP’s standards for methodological rigour and transparency. A smaller number of studies (*n* = 9) were rated as moderate quality, mostly due to limited detail on researcher reflexivity, and ethical considerations. Only one study was low-quality, mainly because of insufficient reporting on the study design, recruitment, data collection, researcher reflexivity, ethical considerations and rigour of data analysis. However, the study was retained in the review for its relevance to the research aim and the insights it provided into the topic of peer involvement, despite the methodological limitations.

Across the dataset, some recurring methodological gaps were identified, including under reporting of researcher reflexivity, and ethical considerations. While quality appraisal did not weight findings, it provides important context for the overall strength of studies. Although emerging research has begun to explore ethical considerations in peer work (61, 63) further study is required. Improved reporting of ethical considerations and researcher reflexivity would enhance the credibility and transparency of future research in this area.

Many studies offered limited reporting on participants’ ethnicity, which was often attributed by authors to small sample sizes and concerns about anonymity. While understandable, the absence of demographic information makes it difficult to explore how experiences of peer involvement in mental health services may vary across ethnic groups. For example, Memon et al. (65) report that individuals from non-white British groups (in the UK often referred to as ‘ethnic minorities’) are less likely to access mental health support, while NHS Digital (66) highlights disproportionately high detention rates under the Mental Health Act among Black populations (66). These patterns suggest that ethnicity can shape both access to and experiences of mental health services, yet these patterns are underexamined in the current peer involvement literature. Greater demographic transparency is needed to ensure more inclusive and representative research in this area.

### 4.2 Strengths of this review

The COM-B model and TDF are well-established frameworks to understand the components of all behaviour, on the principal that the target behaviour is defined (20, 21, 67). A strength of using this theoretical framework is that it enabled a broader understanding of the components that can influence behaviour; capabilities, opportunities, and motivation, but also the influential factors that the theoretical domains highlighted.

Understanding the peer perspective and their reported barriers and facilitators to peer involvement, helps to gain an insight from a real-world perspective, rather than a theoretical one. The inclusion of qualitative studies in this review provided the ability to capture the thoughts, feelings, and experiences that participants had described, to capture a deeper understanding of peer involvement (68).

### 4.3 Limitations of this review

The COM-B model, and the TDF do have some limitations. The fourteen domains on the TDF often overlapped with each other. It was identified in this review, that domains such as knowledge and skills overlapped, as well as identity and social influences. The model and theoretical framework can identify some social influences, however, cannot explain the wider context of the reasons why a behaviour may be influenced, such as cultural background or age (69). Additionally, a limitation of this review is that none of the authors have lived experience of mental health recovery or peer support. Although the authors have professional experience working with peer support roles, the lack of lived experience among the team may limit the insider perspective in interpreting the findings.

A further limitation relates to the geographical distribution of the included studies in this review. Approximately 70% of the included studies were originally conducted in English speaking countries and all studies were undertaken in high income settings. Although there were a small number of studies that were from non-English-speaking contexts, there was a lack of lower- or middle-class representation. As lived experience involvement and PPI practices can vary amongst different cultural and service contexts, the geographical distribution in this review may reduce the extent to which the findings can be applied across varied health systems and sociocultural contexts.

Another limitation of this review is the age criterion defining adulthood as 18 and over, reflecting a Western legal definition. In some cultures, individuals under 18 may be considered adults with similar roles and responsibilities (70). Excluding these groups may omit insights into barriers and facilitators to peer involvement in mental health services, especially in cultures where adulthood begins earlier. Future research should consider culturally diverse definitions of adulthood in the inclusion criteria.

Modest inter-rater reliability was observed during the study screening process. All disagreements were resolved collaboratively, but the lower initial agreement rate may reflect varying experience levels, underscoring the need for clear guidance and consistent application of the eligibility criteria. A further limitation is that the review synthesised a wide variety of peer roles under the broad category of ‘peer involvement.’ Role-specific differences were not consistently reported across studies, with only some studies reporting on specific responsibilities peers’ held. This therefore limited the ability to explore how barriers and facilitators may vary by role or context.

Finally, a formal GRADE-CERQual assessment was not conducted, due to resource constraints, in this review. Applying GRADE-CERQual in future research could strengthen confidence in the synthesised findings.

### 4.4 Implications for practice

The review indicates that peers experience stigma and discrimination from staff in mental health services, impacting their capability, opportunity, and motivation to be involved, raising clear implications for practice. These barriers, rooted in stigma, affected peers’ confidence, emotional wellbeing, and inclusion in staff teams, reducing both their effectiveness and motivation to stay involved in mental health services.

Embedding lived experience across policies, staff training and leadership is widely recommended, with NHS England emphasising peer leadership and formal peer role integration within service models (71). Organisations should review recruitment, training and supervision frameworks to support inclusive practice and formally recognise peer roles. Clear job descriptions and recruitment processes can improve role clarity and status, addressing peers’ experiences of being seen as ‘unglamorous assistants’ rather than valued team members (39).

Expanding opportunities for peers into diverse roles, including leadership, may empower individuals and strengthen their professional identity, helping to ease tensions between lived experience and professional boundaries (72). It also enables fuller use of peers’ knowledge and skills to embed lived experience meaningfully within services. Regular supervision, debriefing and emotional support are essential to manage the emotional challenges described by peers, such as overwhelm and exhaustion (50, 73).

Protecting peers’ autonomy around self-disclosure is also crucial. This review found instances where peers felt pressured to share personal experiences and were left feeling devalued and stigmatised. Policies should safeguard peers’ rights to choose when, how or whether to disclose lived experience.

Anti-stigma interventions in the workplace have been shown to reduce prejudice and discrimination toward people with mental health conditions (74). Embedding these interventions, especially when led or co-facilitated by peers, through workshops, training, or open dialogue, may foster a culture of respect and inclusivity. Peer-led interventions have been found to reduce self-stigma and stigma pressure while empowering peers (75). This review found that when peers influenced organisational culture, challenged discriminatory practices, and promoted trauma-informed care, services became more inclusive and respectful of lived experience.

### 4.5 Future research

Future research on the barriers and facilitators to peer involvement, in mental health services, could explore the perspective of service users and other staff members to further understand the wider systemic context. Furthermore, there was limited information reported on minoritised groups partaking in peer involvement. Further research could explore the barriers and facilitators to peer involvement from the perspective of minoritised groups, including disability, sexual orientation, and ethnicity.

## 5 Conclusion

The findings from this review identified key barriers and facilitators to peer involvement in mental health services. A barrier to peers’ capability to be involved related to the wider staff teams lack of knowledge about the peer role. The conflict between the professional peer role and identity impacted peers’ motivation, positively and negatively, to remain involved. Further barriers to peer involvement were a lack of social opportunities, including limited social support and a power imbalance between peers and other staff. The findings in this review add nuance to the notion that peer involvement reduces stigma and discrimination. While peer involvement can reduce stigma for service users and contribute to shifts in organisational culture, it can also lead to increased stigma and discrimination experienced by peers themselves, particularly from staff and within the organisational context.

## Data Availability

The original contributions presented in the study are included in the article/supplementary material, further inquiries can be directed to Selina Shaw, selinashaw31@gmail.com.
