# Supplementary material for: A systematic review of the barriers and facilitators to lived experience involvement in mental health services
Source: Front Public Health. 2026 Jan 23;13:1737709. doi: 10.3389/fpubh.2025.1737709 (PMC12875955; doi:10.3389/fpubh.2025.1737709)
Supplement: Supplementary file 1 [file Table_1.docx]

Supplementary material

***Table 1: Table of the Summary of the aims, study design, methodology, and data analysis of the included articles.***

| **Study** | **Country** | **Author-identified aims** | **Study design** | **Methodology** | **Data analysis** |
| --- | --- | --- | --- | --- | --- |
| Ben-Dor, *et al*. (2024) | Israel | This study sought to understand perspectives and experiences regarding peers’ SD (self-disclosure) among different stakeholders in mental health services. | Qualitative research approach | Interviews and focus groups | Thematic analysis |
| Brenisin, Padilla, and Breen (2023) | United Kingdom | The purpose of this paper is to provide a particular insight into what it feels like being discharged from psychiatric care from a peer support workers perspective, how may support be improved post-discharge and what factors might impact the potential for readmission into inpatient care. | Descriptive phenomenological design | Semi-structured interview | Interpretative phenomenological analysis |
| Simpson, Oster, and Muir‐Cochrane (2018) | United Kingdom | In the present study, we explored the evolution of peer support workers’ occupational identities. | Qualitative study | Focus groups | Theoretical thematic analysis |
| Vandewalle, *et al.* (2017) | Belgium | The aim of the present study was to develop a conceptual framework representing the driving forces of peer workers to fulfil their position in mental health-care systems. | Qualitative research design | Semi-structured interview | Grounded theory |
| Reeves, *et al.* (2024) | Australia | This research sought to explore the experiences of peer support workers integrating into mental health teams and identify organisational actions to facilitate successful recognition, integration and acceptance by colleagues; therefore, promoting sustainability of the peer support role. | Qualitative descriptive study design | Semi-structured interview | Thematic analysis and qualitative content analysis |
| Janoušková, *et al.* (2022) | Czech Republic | The aim of this study was to explore the characteristics of the PSW role and identify possible conflicts associated with these roles as they are experienced and perceived by peer support workers. | Empirical qualitative study | Semi-structured interview | Interview transcripts were analysed with standard qualitative methods (specific method not specified) |
| Kessing (2021) | Denmark | This paper explores the broader context in which the peer workers navigate and the concrete outcomes and everyday issues that exist at the individual level. | Qualitative study | Semi-structured interview | Coding (specific data analysis method not specified) |
| Storm, *et al.* (2020) | United States | To explore the potential of peer support specialists in community mental health centres and as a means to improve coordination of physical health and mental health services for people with a serious mental illness. | Qualitative study | Semi-structured interview | Thematic analysis |
| Chisholm and Petrakis (2020) | Australia | The study aimed to examine the perspectives of peer workers about ROP (recovery-oriented practice) pre-implementation within one service. | Social constructivist exploratory study | Focus group using semi-structured questions | Thematic analysis |
| Ehrlich, *et al.* (2020) | Australia | Aimed to explore the implementation of a newly formed community-based mental health team. | A cross-sectional qualitative study design | Semi-structured interview | Thematic analysis |
| Oborn, *et al.* (2019) | United Kingdom | What forms of knowledge and expertise do peer workers develop and how they do use them in enacting mental healthcare practices? | Comparative case study design | Semi-structured interview | Complementary thematic and framework approach |
| Gillard, *et al.* (2014) | United Kingdom | This paper aims to model the change mechanisms underlying peer worker interventions. | Qualitative, comparative case study | In-depth interviews | Grounded theory |
| Berry, Hayward, and Chandler (2011) | United Kingdom | The aim of the current evaluation was to use qualitative methodology to further explore the integration of peer support workers into existing mental health teams in the UK. | Qualitative study/evaluation | Semi-structured interview | Thematic analysis |
| Tang, *et al*. (2022) | China | This qualitative study investigated peer supporters’ (PS) perspectives of their roles and experiences of rendering formal peer support to community-dwelling older adults at risk of or living with depression in Hong Kong. | Qualitative design | Focus group using semi-structured questions | Thematic analysis |
| Griffiths and Hancock-Johnson (2017) | United Kingdom | The purpose of this paper is to report the experience and impact of paid staff who are employed to use their lived experience of mental health issues and service use within a secure mental health provider. | Qualitative design | Semi-structured interview | Thematic analysis |
| Holley, Gillard, and Gibson (2015) | United Kingdom | New peer worker roles are being introduced into mental health services internationally. This paper addresses a lack of research exploring issues of risk in relation to the role. | Comparative, qualitative case study | In-depth interviews | Grounded analysis |
| Cleary, *et al.* (2018) | Australia | The aim of this study was to explore the challenges faced by peer support workers when involving mental health consumers in decision-making about their care and the strategies they employed to overcome these challenges so as to improve mental health consumers’ participation in decision-making and recovery. | Qualitative design | Semi-structured interview | Thematic analysis |
| Rebeiro Gruhl, LaCarte, and Calixte (2015) | Canada | To examine the role of the peer support worker (PSW), along with the challenges and benefits, and to understand why the PSW is not more integrated within mainstream services. | Sequential, exploratory, mixed-methods design | Focus group | Thematic analysis |
| Dyble, Tickle, and Collinson (2014) | United Kingdom | The purpose of this paper is to explore the lived experience of NHS employed peer support workers’ transition from their own experiences of mental health problems to provide a service to support individuals with their mental health problems. | Qualitative design | Semi-structured interview | Interpretative phenomenological analysis |
| Beveridge, *et al*. (2019) | Australia | The present study evaluated the feasibility and preliminary efficacy of a PMP (peer mentor programme) for individuals with EDs in improving symptomatology and quality of life. | Mixed methods design | Semi-structured interview | Thematic analysis |
| Wyder, *et al.* (2020) | Australia | This study explores the experience of paid peer support workers integrated within a clinically operated community-based residential rehabilitation service for people diagnosed with a mental health disorder experiencing challenges living independently in the community. | Qualitative design | Diaries | A general inductive approach |
| Kivistö, *et al.* (2023) | Finland | This qualitative study examines the facilitators and challenges of integrating Experts by Experience (EbyE) activity in mental health services in the public sector from the perspective of mental health professionals and trained EbyE. | Qualitative design | Focus group interviews | Abductive content analysis |
| Pérez-Corrales, *et al*. (2019) | Spain | This study sought to explore the views and experiences of a group of people with severe mental disorders (SMDs) who performed volunteer services. | Qualitative phenomenological study | In-depth interviews | Thematic analysis |
| Cabral, *et al.* (2013) | United States | This qualitative study explored how the peer specialist role is defined across different stakeholder groups, the expectations for this role and how the peer specialist is utilised and integrated across different types of mental health services. | Qualitative design | Interviews and focus groups | Consensus coding approach |
| Debyser, *et al*. (2019) | Belgium | This study aims to understand how mental health peer workers experience their transition and how it affects their view of themselves and their direct working context. | Qualitative design | Semi-structured interviews | Grounded theory |
| Soronen (2024) | Finland | This study was conducted to examine the construction of social identity among mental health experts by experience working in Finnish municipal mental healthcare services. | Qualitative design | Focus group | Thematic analysis |
| Moran (2017) | United States | To enhance the PP (peer providers) work role by (1) conceptualizing PPs’ optimal views and experiences of their work role and (2) presenting a prototype self-report measure to assess PP optimal expression in MH services. | Participatory research | Semi-structured interviews and life story interviews | Grounded theory |
| Hancock, *et al*. (2022) | Australia | To understand the impacts and outcomes of the Peer-STOC (supported transfer of care) program on service users from three stakeholder perspectives: service users themselves, peer worker service providers, and other mental health workers and clinicians interfacing with the program. | Qualitative design | In-depth interviews | Thematic analysis |
| Debyser, *et al*. (2018) | Belgium | The aim of this study was to clarify and understand these self-perceptions in order to identify the specificity and potential complementarity of both roles. | Qualitative descriptive research design | Critical incident methodology / personal case reports | Clusters and codes (specific analysis not specified) |
| Poremski, *et al*. (2022) | Singapore | Seeks to determine how peer support roles change as peer support specialists’ positions within organisations and departments mature. | Longitudinal Qualitative (from a larger mixed- methods quasi-experimental study | Repeated qualitative interviews | Resembled a constant comparative approach commonly, but not exclusively, used in grounded theory |
| Gray, Davies, and Butcher (2017) | Australia | The study sought to better understand the workplace environment into which peer support workers were expected to integrate. | Exploratory case study of the organisation | Semi-structured interviews and workplace diaries | Thematic analysis |
| Gillard, *et al*. (2015) | United Kingdom | Aim to establish whether organisational conditions supporting adoption of new peer worker roles in England apply across all mental health providers (or provider partnerships), or whether there are implementation issues that are specific to particular organisational contexts. | Comparative case study design | Structured interview | Thematic and framework approach |
| Gillard, *et al*. (2013) | United Kingdom | Research aim of identifying the organisational benefits and challenges of introducing Peer Worker roles in mental health services. | Secondary analysis of in-depth qualitative interview data. The primary study was a mixed method organisational study | Semi-structured interviews | Grounded theory |
