# Supplementary material for: A systematic review of the barriers and facilitators to lived experience involvement in mental health services
Source: Front Public Health. 2026 Jan 23;13:1737709. doi: 10.3389/fpubh.2025.1737709 (PMC12875955; doi:10.3389/fpubh.2025.1737709)
Supplement: Supplementary file 2 [file Table_2.docx]

***Table 2: Table of participant characteristics, including number of participants, age, gender, educational level, diagnosis, role and activities, length of commitment, and the type of organisation, for the included articles (NS = not specified)***

| **Study** | **Number of participants** | **Age** | **Gender (F = female,  M= male)** | **Educational level** | **Previous experience of mental health (i.e. diagnosis)** | **Role** | **Role activities** | **Length of commitment** | **Type of organisation** |
| --- | --- | --- | --- | --- | --- | --- | --- | --- | --- |
| Ben-Dor, *et al*. (2024) | 13 | M = 40.5 | 9F, 4M | NS | NS | Peer workers | NS | NS | Mental health hospitals, and community rehabilitation |
| Brenisin, Padilla, and Breen (2023) | 4 | NS | 3F, 1M | NS | NS | Peer support workers | Individual patient support | NS | Medium/low secure mental health wards |
| Simpson, Oster, and Muir‐Cochrane (2018) | 8 | Mean age = 41.6 years | 5M, 3F | NS | NS | Peer support workers | NS | 12 weeks | Mental health wards to the community (transition service) |
| Vandewalle, *et al.* (2017) | 14 | 30-40 (N=4), 41-50 (N=2), 51-60 (N=7) and over 60 (N=1). | 8F, 6M | Master (N=4), Undergraduate (N=5) and Secondary school (N=5) | NS | Peer workers | Group facilitators, one to one support, admin, MDT working. | NS | Residential and community mental healthcare |
| Reeves, *et al.* (2024) | 18 | NS | 12F, 6M | NS | NS | Peer support workers | Group work, one to one support. | 3 months - 20 years | Inpatient and community mental health services |
| Janoušková, *et al.* (2022) | 15 | 31-56 years | 8F, 7M | NS | NS | Peer support workers | NS | 4 months - 7 years | Psychiatric hospitals and community mental health services |
| Kessing (2021) | 22 | NS | NS | Varying levels - some peer workers had academic degrees | Post-traumatic stress syndrome, bipolar affective disorder and depression | Peer workers | One to one and group work. Staff meetings and building networks for service users. | NS | Mental health wards |
| Storm, *et al.* (2020) | 5 | NS | 5F | NS | NS | Peer support specialists | Representing the service user, serving as members of the clinical team, | NS | Community mental health centres |
| Chisholm and Petrakis (2020) | 8 | NS | 7F, 1M | NS | NS | Peer workers | NS | 0-20 years | Clinical mental health service |
| Ehrlich, *et al.* (2020) | 5 | NS | NS | NS | NS | Peer support workers | NS | NS | Community based mental health team |
| Oborn, *et al.* (2019) | 22 | NS | NS | NS | NS | Peer workers | NS | NS | Inpatient, community, and Black and Minority Ethnic (BME)- specific services |
| Gillard, *et al.* (2014) | 12 | NS | NS | NS | NS | Peer support workers | NS | NS | General adult mental health |
| Berry, Hayward, and Chandler (2011) | 2 | NS | NS | NS | NS | Peer support specialists | NS | NS | NHS mental health trust |
| Tang, *et al*. (2022) | 27 | 54-74 | 21 F, 6M | NS | NS | Peer supporters | One to one support, group facilitators, speaking at events. | Minimum 12 months | Community mental health support for older adults at risk of depression |
| Griffiths and Hancock-Johnson (2017) | 3 | NS | NS | NS | NS | Lived experience workers | Training, one to one support, service design and development. | NS | Secure mental health setting |
| Holley, Gillard, and Gibson (2015) | 12 | NS | NS | NS | NS | Peer support workers | NS | NS | General adult mental health |
| Cleary, *et al.* (2018) | 6 | NS | NS | NS | NS | Peer support workers | NS | NS | Psychiatric hospitals and/or community mental health |
| Rebeiro Gruhl, LaCarte, and Calixte (2015) | 25 | NS | NS | NS | NS | Peer support workers | Admin, one to one support, MDT working, supervision. | NS | Mainstream mental health services |
| Dyble, Tickle, and Collinson (2014) | 7 | 22-59 | 5F, 2M | NS | Psychosis (n = 5); bipolar; stress-related illness; depression and anxiety; and anorexia nervosa. Never received a diagnosis (n = 1) and did not wish to disclose diagnosis (n = 1) | Peer support workers | NS | NS | Adult mental health services |
| Beveridge, *et al*. (2019) | 15 | 23-39 | 14F, 1M | Tertiary education (n = 6), vocational course/diploma (n = 2) secondary school (n = 1) (six unreported) | Eating disorder | Mentors | One to one support. | NS | Eating disorder services |
| Wyder, *et al.* (2020) | 36 | NS | NS | NS | NS | Peer workers | NS | NS | Community- residential rehabilitation service |
| Kivistö, *et al.* (2023) | 13 |  | 3M, 10F | NS | NS | Expert by experience | Lecturing, one to one support, service development, and group facilitators. | 2-10 years | Mental health services |
| Pérez-Corrales, *et al*. (2019) | 23 | mean = 47 years | 16M, 7F | NS | 11 participants: F20.0: paranoid schizophrenia. 3 participants: F31: bipolar disorder. 2 participants: F20.5: residual schizophrenia. 2 participants: F25: schizoaffective disorder. 2 participants: F20.9: unspecified schizophrenia. 1 participant: F20.6: simple schizophrenia. 1 participant: F33: recurrent depressive disorder. 1 participant: F28: other psychotic disorder | Volunteers | NS | NS | Community public mental health services |
| Cabral, *et al.* (2013) | 44 | NS | 18M, 26 F | NS | NS | Mental health peer specialists | NS | NS | Different types of mental health services |
| Debyser, *et al*. (2019) | 17 | 30-39 (N=3), 40-49 (N=4), 50-59 (N=9), over 60 (N=1) | 7M, 10F | Unknown (N=1), Secondary education (N=5), Higher education (N=6),  University education (N=5) | Mood disorders, trauma-related problems, personality disorders, substance-related problems, anxiety disorders, or combinations of previous diagnoses | Mental health peer workers | Group facilitators, one to one support, consultation, informing policies and guidance. | Less than 12 months - 48 months | Mental healthcare organisations |
| Soronen (2024) | 18 | NS | NS | Vocational education, and higher education (specific number NS) | NS | Expert by experience | NS | 1-20 years | Mental health units |
| Moran (2017) | 25 | 23-63 | 14F, 11M | 56% had graduated college | Diagnosis of a psychiatric Axis I condition, such as schizophrenia spectrum disorders or affective disorders | Peer provider | Group facilitators, one to one support. | 8 months - 15 years | Community rehabilitation services and psychiatric wards |
| Hancock, *et al*. (2022) | 22 | 18-29 (n-3), 30-44 (n=7), 45-64 (n= 12) | 11 F, 7 M, 4 non-binary/other identity | NS | NS | Peer workers | NS | 1-5 years | Transition from mental health care to community |
| Debyser, *et al*. (2018) | 8 | 30-39 (n= 5), 40-49 (n= 2), >50 (n= 1) | 4M, 4F | University education (n=2), Professional bachelor (n= 5), Lower secondary education (n= 1) | NS | Mental health peer workers | NS | 1-5 years | Community mental health, psychiatric hospitals |
| Poremski, *et al*. (2022) | 10 | Average age 30 years | 6F, 4M | Post-secondary education | NS | Peer support specialists | NS | NS | Tertiary psychiatric care |
| Gray, Davies, and Butcher (2017) | 2 | NS | NS | NS | NS | Peer support workers | Group facilitator, small client caseload. | NS | Community mental health services |
| Gillard, *et al*. (2015) | 12 | NS | NS | NS | NS | Peer support workers | NS | NS | General adult mental health |
| Gillard, *et al*. (2013) | 15 | NS | NS | NS | NS | Peer workers | Group co-facilitator, trainers, recovery planning | NS | Self-care intervention service |
