# Supplementary material for: A systematic review of the barriers and facilitators to lived experience involvement in mental health services
Source: Front Public Health. 2026 Jan 23;13:1737709. doi: 10.3389/fpubh.2025.1737709 (PMC12875955; doi:10.3389/fpubh.2025.1737709)
Supplement: Supplementary file 3 [file Table_3.docx]

***Table 3: Matrix of COM-B and TDF themes within each paper***

|  | Capability | | | | Opportunity | | Motivation | | | | | | | |
| --- | --- | --- | --- | --- | --- | --- | --- | --- | --- | --- | --- | --- | --- | --- |
| Paper | Psychological skills | Knowledge | Behaviour regulation | Memory, attention, and decision-making | Environmental context and resource | Social influences | Reinforcement | Emotions | Intentions | Goals | Social, professional role and identity | Optimism | Beliefs about capabilities | Beliefs about consequences |
| Ben-Dor, *et al*. (2024) |  | ■ |  |  | ■ | ■ |  |  |  |  | ■ |  | ■ | ■ |
| Brenisin, Padilla, and Breen (2023) |  | ■ |  |  |  |  | ■ |  |  |  |  |  |  |  |
| Simpson, Oster, and Muir‐Cochrane (2018) | ■ | ■ |  |  | ■ | ■ |  | ■ |  |  | ■ |  | ■ | ■ |
| Vandewalle, *et al.* (2017) |  |  |  |  | ■ | ■ |  | ■ |  | ■ | ■ |  | ■ | ■ |
| Reeves, *et al.* (2024) |  | ■ |  |  | ■ | ■ |  |  |  |  | ■ |  | ■ |  |
| Janoušková, *et al.* (2022) | ■ |  |  |  | ■ | ■ |  |  |  |  | ■ |  | ■ |  |
| Kessing (2021) | ■ | ■ |  |  | ■ |  |  |  |  |  | ■ |  |  |  |
| Storm, *et al.* (2020) | ■ |  |  |  |  |  |  |  |  | ■ | ■ |  | ■ |  |
| Chisholm and Petrakis (2020) | ■ | ■ |  |  | ■ | ■ |  |  |  |  | ■ |  |  |  |
| Ehrlich, *et al.* (2020) | ■ |  |  |  | ■ |  |  | ■ |  |  | ■ |  | ■ | ■ |
| Oborn, *et al.* (2019) | ■ | ■ |  |  | ■ | ■ |  |  |  |  |  |  |  |  |
| Gillard, *et al.* (2014) | ■ | ■ |  |  | ■ | ■ |  |  |  |  | ■ |  |  |  |
| Berry, Hayward, and Chandler (2011) | ■ | ■ |  |  | ■ | ■ |  |  |  |  | ■ |  | ■ |  |
| Tang, *et al*. (2022) | ■ | ■ |  | ■ |  | ■ | ■ | ■ |  |  | ■ |  |  |  |
| Griffiths and Hancock-Johnson (2017) |  | ■ |  |  | ■ | ■ |  | ■ |  |  | ■ |  | ■ |  |
| Holley, Gillard, and Gibson (2015) |  | ■ |  |  | ■ | ■ |  | ■ |  |  | ■ |  |  | ■ |
| Cleary, *et al.* (2018) | ■ | ■ |  | ■ | ■ | ■ |  |  |  |  | ■ |  |  |  |
| Rebeiro Gruhl, LaCarte, and Calixte (2015) | ■ | ■ |  |  | ■ |  | ■ | ■ |  |  | ■ |  | ■ |  |
| Dyble, Tickle, and Collinson (2014) |  |  |  |  | ■ | ■ |  |  |  |  | ■ |  | ■ |  |
| Beveridge, *et al*. (2019) |  | ■ |  |  |  | ■ |  | ■ |  |  |  |  | ■ |  |
| Wyder, *et al.* (2020) | ■ | ■ |  |  |  |  |  |  |  |  | ■ |  | ■ |  |
| Kivistö, *et al.* (2023) | ■ |  |  |  | ■ | ■ |  |  |  |  | ■ |  |  |  |
| Pérez-Corrales, *et al*. (2019) |  |  |  |  |  |  | ■ |  |  |  | ■ |  | ■ |  |
| Cabral, *et al.* (2013) | ■ | ■ |  |  | ■ | ■ |  |  |  |  | ■ |  |  |  |
| Debyser, *et al*. (2019) | ■ |  |  |  | ■ | ■ | ■ |  |  |  | ■ |  | ■ |  |
| Soronen (2024) | ■ |  |  |  |  | ■ | ■ |  |  | ■ | ■ |  | ■ |  |
| Moran (2017) | ■ | ■ |  |  | ■ | ■ |  |  |  | ■ | ■ |  |  |  |
| Hancock, *et al*. (2022) | ■ | ■ |  |  |  | ■ |  |  |  |  |  |  |  |  |
| Debyser, *et al*. (2018) | ■ |  |  |  |  | ■ |  |  |  |  | ■ |  | ■ |  |
| Poremski, *et al*. (2022) |  |  |  |  | ■ | ■ |  |  |  |  | ■ |  |  | ■ |
| Gray, Davies, and Butcher (2017) | ■ | ■ |  |  |  | ■ |  |  |  |  | ■ |  |  |  |
| Gillard, *et al*. (2015) |  | ■ |  |  | ■ | ■ |  |  |  |  | ■ |  | ■ |  |
| Gillard, *et al*. (2013) |  | ■ |  |  | ■ | ■ | ■ | ■ |  | ■ | ■ |  |  | ■ |

■ Indicates presence of the theme in the paper
